# Association of education attainment, smoking status, and alcohol use disorder with dementia risk in older adults: a longitudinal observational study

**DOI:** 10.1186/s13195-024-01569-7

**Published:** 2024-09-18

**Authors:** Huilin Tang, C. Elizabeth Shaaban, Steven T. DeKosky, Glenn E Smith, Xia Hu, Michael Jaffee, Ramzi G. Salloum, Jiang Bian, Jingchuan Guo

**Affiliations:** 1 Department of Pharmaceutical Outcomes and Policy, University of Florida College of Pharmacy, Gainesville, FL 32606 USA; 2 Department of Epidemiology, School of Public Health, University of Pittsburgh, Pittsburgh, PA USA; 3 Alzheimer’s Disease Research Center, University of Pittsburgh, Pittsburgh, PA USA; 4 Department of Neurology and McKnight Brain Institute, College of Medicine, University of Florida, Gainesville, FL USA; 5 1Florida Alzheimer’s Disease Research Center (ADRC), University of Florida, Gainesville, FL USA; 6 Department of Clinical and Health Psychology, College of Public Health and Health Professions, University of Florida, Gainesville, FL USA; 7 DATA Lab, Department of Computer Science, Rice University, Texas, USA; 8 Department of Health Outcomes and Biomedical Informatics, College of Medicine, University of Florida, Gainesville, FL USA; 9 Center for Drug Evaluation and Safety, University of Florida, Gainesville, FL USA

**Keywords:** Social and behavioral determinants of health, College education, Smoking, Alcohol use disorder, All-cause dementia

## Abstract

**Background:**

Previous research on the risk of dementia associated with education attainment, smoking status, and alcohol use disorder (AUD) has yielded inconsistent results, indicating potential heterogeneous treatment effects (HTEs) of these factors on dementia risk. Thus, this study aimed to identify the important variables that may contribute to HTEs of these factors in older adults.

**Methods:**

Using 2005–2021 data from the National Alzheimer’s Coordinating Center (NACC), we included older adults (≥ 65 years) with normal cognition at the first visit. The exposure of interest included college education or above, current smoking, and AUD and the outcome was all-cause dementia. We applied doubly robust learning to estimate risk differences (RD) and 95% confidence intervals (CI) between exposed and unexposed groups in the overall cohort and subgroups identified through a decision tree model.

**Results:**

Of 10,062 participants included, 929 developed all-cause dementia over a median 4.4-year follow-up. College education or above was associated with a lower risk of all-cause dementia in the overall population (RD, -1.5%; 95%CI, -2.8 to -0.3), especially among the subpopulations without hypertension, regardless of the *APOE4* status. Current smoking was not related to increased dementia risk overall (2.8%; -1.5 to 7.2) but was significantly associated with increased dementia risk among men with (21.1%, 3.1 to 39.1) and without (8.4%, 0.9 to 15.8) cerebrovascular disease. AUD was not related to increased dementia risk overall (2.0%; -7.7 to 11.7) but was significantly associated with increased dementia risk among men with neuropsychiatric disorders (31.5%; 7.4 to 55.7).

**Conclusions:**

Our studies identified important factors contributing to HTEs of education, smoking, and AUD on risk of all-cause dementia, suggesting an individualized approach is needed to address dementia disparities.

**Supplementary Information:**

The online version contains supplementary material available at 10.1186/s13195-024-01569-7.

## Background

Dementia, characterized by a loss of cognitive function, affects independence and daily activities [1]. It remains a major public health problem that impacts about 55 million people worldwide [2]. In the United States (US), about 6.5 million adults had a diagnosis of dementia in 2022 and this number is expected to rise to 14 million by 2060 [3, 4]. Alzheimer’s disease (AD), the most common cause of dementia, is ranked as the sixth leading cause of death in the US [3]. Although the fundamental etiology of dementia is yet to be fully elucidated, it is posited to be caused by a combination of genetic, health behavior, and environmental determinants [5, 6]. Currently, there is no cure for dementia, but there are interventions that can help manage the symptoms and have small disease-modifying effects [7, 8].

Increasing evidence has shown that social and behavioral determinants of health (SBDH) have an important influence on health outcomes, accounting for 30–50% of outcomes [9, 10]. Educational attainment, smoking status, and alcohol use disorder (AUD) have been identified as the three major factors that may contribute to the development of dementia, although their association remains inclusive [11]. Educational attainment has long been hypothesized to play a protective role against cognitive decline and dementia [12, 13]. This protective effect is often attributed to the concept of cognitive reserve, which posits that higher levels of education may enhance neural networks and cognitive strategies, potentially delaying the clinical manifestation of dementia symptoms. Conversely, low levels of formal education or a complete lack thereof have been associated with an increased risk of dementia [14]. However, the mechanisms underlying this relationship and the extent of its impact across different populations require further elucidation. The relationship between smoking and dementia risk has been the subject of numerous studies, yielding somewhat inconsistent results [15–17]. These indicated a possible heterogeneity in treatment effects between smoking and risk of dementia. Alcohol consumption and its impact on dementia risk presents another area of significant interest and complexity in the population study [18, 19], with a particular focus on the potentially detrimental effects of AUD [20].

Despite the wealth of research in these areas, significant gaps in our understanding persist, particularly regarding how these risk factors may differentially affect various subgroups within the population. Heterogeneous treatment effects (HTEs) become crucial, as it examines varying treatment effects for individuals or subgroups in a population. Doubly robust learning is a powerful data-driven approach for estimating HTEs, enabling a deeper understanding of the intricate relationships between variables and their impact on the outcome. Leveraging this advanced analytical technique, this study aimed to investigate the HTEs of educational attainment, smoking, and AUD on risk of all-cause dementia and elucidate how these factors differentially influence dementia risk across various subgroups within the older population. The findings of this study contribute valuable insights to the field of dementia prevention and risk stratification, potentially informing more targeted and effective public health interventions and clinical strategies.

## Methods

### Data source

This retrospective cohort study was performed using the National Alzheimer’s Coordinating Center (NACC) dataset, which was founded by the National Institute on Aging in 1999 and contains data contributed by the 39 U.S. Alzheimer’s Disease Research Centers (ADRCs). We used the NACC Uniform Data Set (UDS) collected between September 2005 and June 2021 [21]. The UDS contains data collected through a prospective, longitudinal clinical examination by trained clinicians and clinic personnel from participants and their co-participants across the ADRCs. This includes data on personal characteristics, demographics, health behaviors, current health conditions and disease history, medication use, functional abilities, depressive symptoms, and detailed neuropsychological testing. Informed consent forms were approved by the individual ADRCs’ Institutional Review Boards (IRBs), and consent was obtained from participants and study partners before research activities were carried out. Research using the NACC database was also approved by the University of Washington IRB.

### Participant selection and outcome measurement

In this study, we included participants in the analytic sample if they met the following criteria: (1) had ≥ 2 clinical visits at ADRCs between September 2005 and June 2021; (2) were aged ≥ 65 years at baseline (first visit); and (3) had normal cognition at baseline. We excluded participants who took any anti-dementia drug at baseline. The participants were followed until the occurrence of a dementia diagnosis, discontinuation from the study, or completion of the study.

The cognitive diagnosis for each person was determined through clinician judgment or by a multidisciplinary consensus team using The Clinician Diagnosis form in the UDS [21]. Each person was characterized as having “normal cognition”, “impaired not mild cognitive impairment (MCI)”, “MCI”, or “dementia” at the initial visit and each subsequent follow-up visit [22]. The outcome of interest in this study was all-cause dementia. The criteria for diagnosing dementia was based on recommendations by the National Institute on Aging-Alzheimer’s Association workgroups on diagnostic guidelines for Alzheimer’s disease [23].

#### Definition of exposure of interest

The exposure of interest in this study included college education or above (yes vs. no), current smoking (yes vs. no), and AUD (yes vs. no) in the NACC dataset. Smoking status and AUD were extracted from the subject’s health history (UDS Form A5) completed by a clinician based on “*subject/informant report*,* medical records*,* and/or observation*” using the clinician’s best judgment. According to the UDS question, “smoked cigarettes in last 30 days”, the participants were divided in two groups (current smokers vs. others). Participants were also asked about their alcohol use and were classified to have AUD (yes) if they reported recent clinically significant impairment due to alcohol abuse occurring over 12 months manifested in one of the following areas: work, driving, legal, or social.

### Covariates

The covariates (including demographic factors, health behaviors, comorbidities, and genetic factors) that may be related to risk of dementia, were collected, and adjusted for in the study [24–26]. The demographic characteristics included age (≥ 80 vs. < 80 years), sex (male vs. female), race (Black vs. non-Black), ethnicity (Hispanic vs. non-Hispanic), and family history of dementia (yes vs. no). Participants were classified as obese (yes vs. no) based on their body mass index at baseline (≥ 30 kg/m^2^). Comorbidities were classified as the presence vs. absence of self-reported history of diseases like cardiovascular disease (including heart attack/cardiac arrest, angioplasty/endarterectomy/stent, cardiac bypass procedure, pacemaker and/or defibrillator, congestive heart failure, atrial fibrillation, angina, heart valve replacement or repair, and other cardiovascular diseases), cerebrovascular disease (including stroke and transient ischemic attack), neurological diseases (including Parkinson’s disease, other Parkinson’s disease disorders, traumatic brain injury, seizures, and other neurological conditions), neuropsychiatric disorders (post-traumatic stress disorder, bipolar disorder, schizophrenia, depression, anxiety, obsessive-compulsive disorder, developmental neuropsychiatric disorders, and other psychiatric disorders), diabetes, hypercholesterolemia, and hypertension. We also controlled for the *APOE4* gene, the strongest known genetic risk factor for AD and cognitive impairment [26].

### Statistical analysis

We described the demographic and clinical characteristics of the study sample at baseline. Additionally, we also assessed the balance of baseline covariates between exposure and non-exposure groups using standardized mean differences (SMD) before and after the inverse probability of treatment weighting (IPTW). An SMD of ≤ 0.1 was considered to be an imbalance in the baseline covariate [27].

We estimated the conditional average treatment effect (CATE) in terms of the difference in risk of all-cause dementia between the exposed group and non-exposed group following the doubly robust learning framework [28]. In the initial models, aiming to select optimal hyperparameters of each model, we designed two predictive tasks: (1) predicting the probability of having exposure for every subject using the propensity score model and achieving balances of baseline covariate between exposure and non-exposure groups using IPTW; (2) estimating the risk of all-cause dementia for both the exposures of interest and non-exposure group using the outcome regression models. In the final model which combined the above two predictive models, we calculated the doubly robust causal estimate [29]. This model provides a correct estimate even if either the propensity score model or outcome regression is misspecified.

We randomly split the sample into a training (70%) set and a test (30%) set. The training set was used to train the machine learning models with hyperparameter optimization, and the testing set was used to evaluate the models’ prediction performance. In the initial models, we applied logistic regression for the propensity score model and least absolute shrinkage and selection operator-type regularized regression (LASSO) for the outcome regression model. In the final stage model, we estimated the CATEs in the overall cohort and predicted individualized treatment effects (ITEs, treatment effects on the personal level) using SparseLinearDRLearner within EconML package [28]. We measured the model performance using the score based on the final stage loss (a lower score is better) and assessed the out-of-sample score on the testing set. The treatment effect was quantified as the risk difference (RD) with 95% confidence interval (CI) for risk of all-cause dementia between exposed and unexposed groups in each SBDH. To identify the important covariates and discover drivers of heterogeneity, we applied a single decision tree model for the treatment effect. Based on the important covariates, the tree-based model split the participants into subgroups, in which a subgroup of samples responded to treatment differently from other subgroups.

To address the competing risk of death, we conducted a sensitivity analysis by applying a doubly robust estimation of the hazard difference for competing risk data using the R Package “HazardDiff.” [30]. All analyses were performed using SAS version 9.4 (SAS Institute Inc), and Python version 3.7 (Python Software Foundation).

## Results

### Study population

**Fig. 1:**
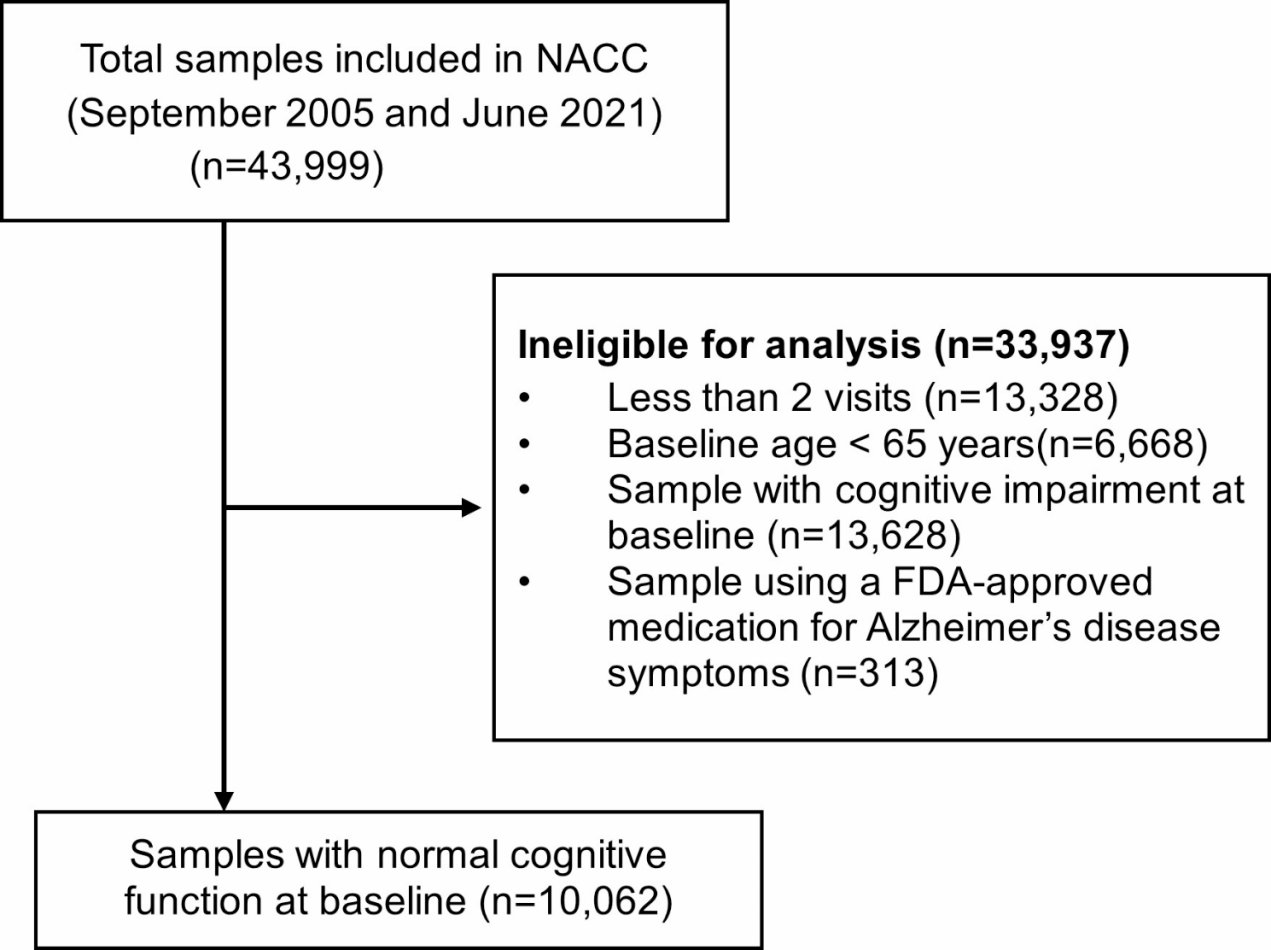
Flowchart of participant selection. NACC, National Alzheimer’s Coordinating Center; FDA, Food and Drug Administration

The flowchart of participant selection based on the inclusion and exclusion criteria is presented in Fig. 1. Of 43,999 participants included in the NACC from September 2005 to June 2021, we included 10,062 participants with normal cognitive function at baseline (at 1st clinical visit) in this cohort study and outlined the reasons for exclusion in Fig. 1. The demographic and clinical characteristics of all participants at baseline are presented in Table 1. The mean age of the sample was 74.9 years, 35.5% were men, 14.2% were Black, and 5.6% were Hispanic. Among all participants, 929 participants (9.2%) developed all-cause dementia over a median follow-up of 4.4 years (Interquartile range, 2.2 to 7.7). Participants who developed dementia were older and were more likely to have a family history of dementia and be *APOE4* carriers.

**Table 1:** Descriptive statistics of the characteristics of participants included in this study (*n* = 10,062)

	NACC participants (free of dementia at baseline)	No dementia development during follow-up	Dementia development during follow-up
No. of participants	10,062	9133	929
**Sociodemographic**			
Age, years, mean(sd)	74.9(7.0)	74.5 (6.8)	79.1(7.0)
Age, ≥ 80 years	2619 (26.0)	2173 (23.8)	446(48.0)
Sex, male %	3570 (35.5)	3237(35.4)	333(35.8)
Educational level, ≥ college education	6390 (63.5)	5838 (63.9)	552(59.4)
Hispanic/Latino ethnicity	559 (5.6)	522(5.7)	37(4.0)
Race, Black	1429 (14.2)	1334(14.6)	95(10.2)
Family history of dementia	5165 (51.3)	4636(50.8)	529(56.9)
*APOE4* carrier	2546 (25.3)	2215(24.3)	331(35.6)
**Health Behaviors**			
Current smoking	341 (3.4)	308(3.4)	33(3.6)
Alcohol use disorder	37 (0.4)	32(0.4)	5(0.5)
**Comorbidities**			
Cardiovascular disease	2537 (25.2)	2276(24.9)	261(28.1)
Cerebrovascular disease	661 (6.6)	562(6.2)	99(10.7)
Neurological diseases	777 (7.7)	721(7.9)	56(6.0)
Neuropsychiatric disorders	2555 (25.4)	2323(25.4)	232(25.0)
Diabetes	1212 (12.1)	1108(12.1)	104(11.2)
Hypercholesterolemia	5156 (51.2)	4713(51.6)	443(47.7)
Hypertension	5271 (52.4)	4754(52.1)	517(55.7)
Obesity	781 (23.3)	2185(23.9)	159(17.1)

Cardiovascular disease consists of heart attack/cardiac arrest, angioplasty/endarterectomy/stent, cardiac bypass procedure, pacemaker and/or defibrillator, congestive heart failure, atrial fibrillation, angina, heart valve replacement or repair, and other cardiovascular diseases;

Cerebrovascular disease included stroke and transient ischemic attack;

Neurological disease involves Parkinson’s disease (PD), other PD disorders, traumatic brain injury, seizures, and other neurological conditions;

Neuropsychiatric disorders include post-traumatic stress disorder, bipolar disorder, schizophrenia, depression, anxiety, obsessive-compulsive disorder, developmental neuropsychiatric disorders, and other psychiatric disorders

### College education or above and the risk of all-cause dementia

**Fig. 2:**
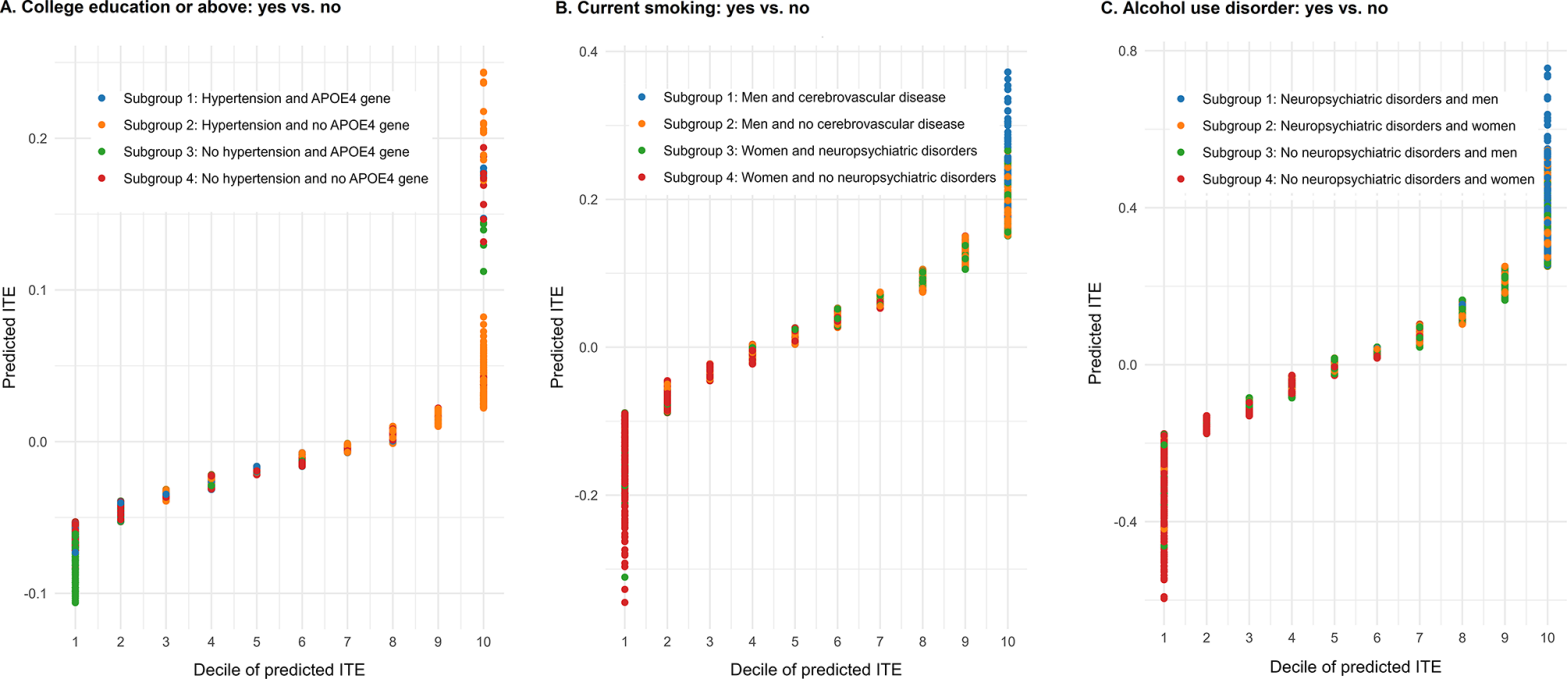
Heterogeneous treatment effects of education attainment (**A**), smoking status (**B**), and alcohol use disorder (**C**) on risk of all-cause dementia. The predicted individualized treatment effect (ITE) was presented as an absolute risk difference in risk of dementia between exposure and non-exposure for each participant (Y-axis). The predicted ITE is divided into 10 groups according to the deciles (X-axis). Different colors represent participants in distinct subgroups identified by the single decision tree model

**Fig. 3:**
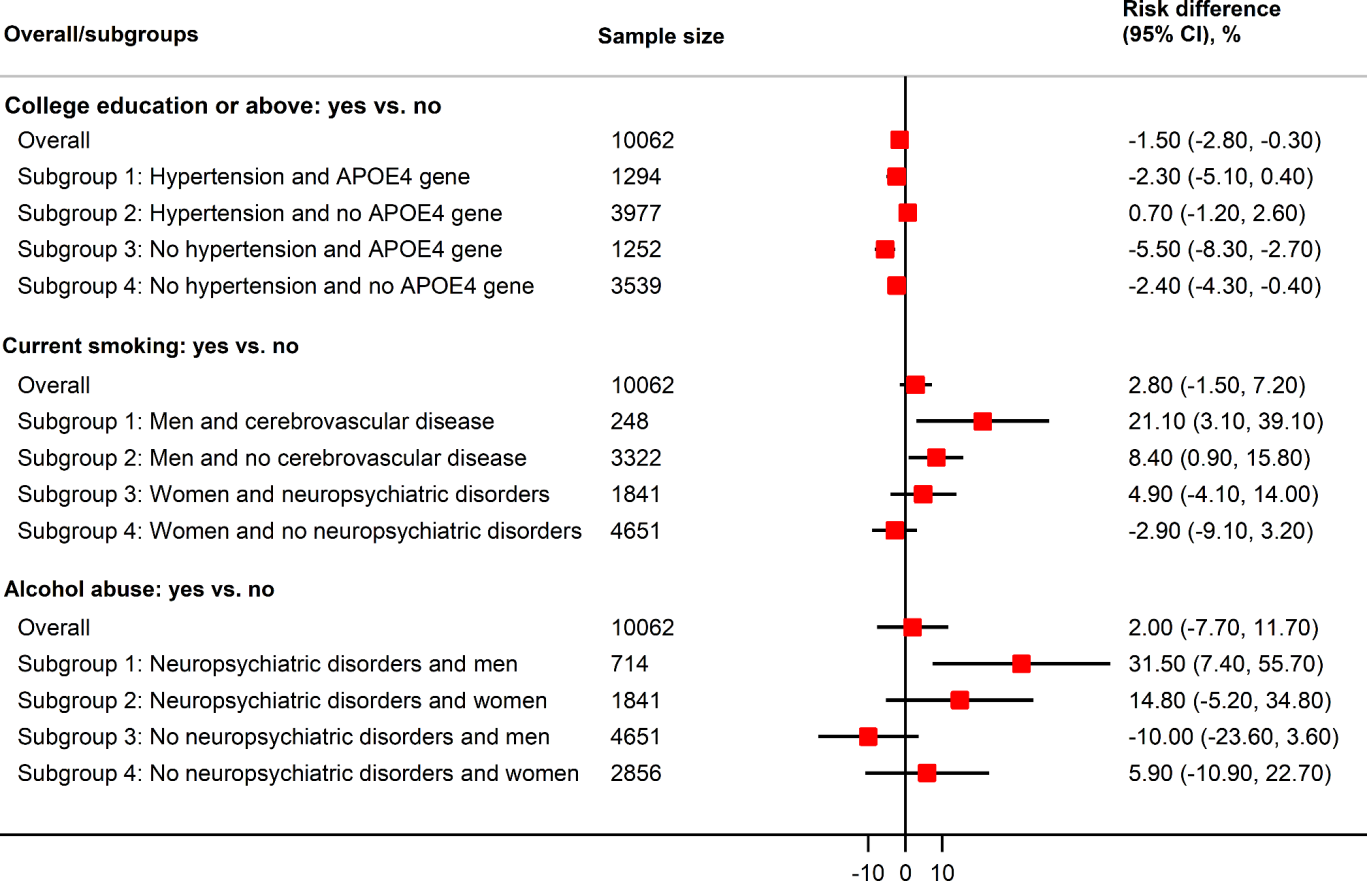
Absolute risk difference in risk of all-cause dementia associated with education attainment (**A**), smoking status (**B**), and alcohol use disorder (**C**) in the overall population and subgroups identified by the single decision tree model. CI, confidence interval

The baseline characteristics of the participants, stratified by college attainment (those with college education or above vs. those without), are presented in Table S1. Among participants with college education or above, they seemed to be younger, having a higher percentage of men, and a lower percentage of Hispanic/Latino ethnicity, Black, current smoker, diabetes, hypertension, and obesity. All covariates were well-balanced after IPTW. The final parameter set of propensity score model and outcome regression model is present in Table S2 and the performance of the final model is presented in Table S3. In the estimation of CATEs, college education or above was associated with a lower risk of all-cause dementia (RD: -1.5%; 95%CI, -2.8% to -0.3%). We estimated the heterogeneous effect of college education or above on risk of all-cause dementia (Fig. 2). Among all participants included, 71.0% had a decreased risk of dementia. The HTE subgroups based on a single decision tree model is presented in Fig. 3 and Figure S1. Having hypertension and the *APOE4* gene were the most important factors. We found a significant decrease in risk of all-cause dementia associated with college education or above among participants without hypertension regardless of the *APOE4* gene with an RD of -5.5% (-8.3% to -2.7%) for those with *APOE4* and − 2.4% (-4.3% to -0.4%) for those without *APOE4*. However, we found no significant decrease in risk among those with hypertension regardless of the *APOE4* gene.

### Current smoking and risk of all-cause dementia

The baseline characteristics of participants by exposure and non-exposure groups for current smoking are presented in Table S4. Among participants with current smoking, they seemed to be younger, having a lower percentage of college education or above, and a higher percentage of Black population, AUD, neuropsychiatric disorders, and hypertension. All covariates were well-balanced after IPTW. The final parameter set of propensity score model and outcome regression model is present in Table S2 and the performance of the final model is presented in Table S3. The final model seemed to be overfitting with a score of 5.2 for the training set and 8.8 for the testing set. In the estimation of CATEs, current smoking was not significantly associated with an increased risk of all-cause dementia (2.8%; -1.5–7.2%). The results based on the heterogeneous effect of current smoking on all-cause dementia risk showed that 39.2% of participants had a decreased risk of dementia (Fig. 2). Male sex, cerebrovascular disease, and neuropsychiatric disorder were the most important covariates identified in the single decision tree model (Fig. 3 and Figure S1). Current smoking was significantly associated with an increased risk of all-cause dementia among men with cerebrovascular disease (21.1%; 3.1–39.1%) and men without cerebrovascular disease (8.4%; 0.9–15.8%). No significant differences were observed in other HTE subgroups (Fig. 3).

### AUD and the risk of all-cause dementia

The baseline characteristics of the participants, stratified by AUD status, are presented in Table S5. Participants with AUD, compared to those without, seemed to be younger, having a lower percentage of Hispanic/Latino ethnicity, Black population, and hypercholesterolemia, and a higher percentage of family history of dementia, current smokers, neurological disorders, neuropsychiatric disorders, diabetes, and hypertension. The covariates were well-balanced after IPTW except for cardiovascular disease, neurological disease, and diabetes. The final parameter set of propensity score model and outcome regression model is present in Table S2 and the performance of the final model is presented in Table S3. The final model seemed to be overfitting with a score of 16.7 for the training set and 22.1 for the testing set. In the estimation of CATEs, there was no association between AUD and risk of all-cause dementia (2.0%; -7.7–11.7%) as compared to no AUD. A heterogeneous effect of AUD on all-cause dementia risk was estimated (Fig. 2). Among all participants included, 47.3% of participants had a decreased risk. Having neuropsychiatric disorders and being male were the most important factors identified in the single decision tree model (Fig. 3 and Figure S1). AUD was significantly associated with an increased risk of all-cause dementia among male participants with neuropsychiatric disorders (RD, 31.5%; 95%CI, 7.4–55.7%). However, no significant association between AUD and all-cause dementia risk in other HTE subgroups was detected (Fig. 3**)**.

### Sensitivity analysis

In the sensitivity analysis when accounting for the competing risk of death, the hazard difference for College education or above, current smoking, and AUD was − 0.3% (-0.6% to -0.1%), 0.7%(0.01–1.5%), and − 0.8% (-2.5–0.9%), respectively.

## Discussion

In this retrospective cohort study of 10,062 older adults from NACC, we found that college education or above was significantly associated with a decreased risk of all-cause dementia while neither current smoking nor AUD was associated with all-cause dementia risk. We identified the most important covariates that may contribute to the HTE subgroups for each exposure. Participants with college education or above had a lower risk of all-cause dementia in those without hypertension regardless of carrying the *APOE4* gene, but not in those with hypertension. Current smoking was shown to be significantly associated with an increased risk of all-cause dementia among men with and without cerebrovascular disease. AUD was significantly associated with an increased risk of all-cause dementia in men with neuropsychiatric disorders.

The relationship between education attainment and risk of all-cause dementia has been widely studied [31–34]. Our study indicated that participants with college education or above had a lower risk for all-cause dementia, which is consistent with prior research [35, 36]. One meta-analysis of 69 prevalence and/or incidence studies showed that lower education was significantly associated with an increased risk of dementia with an odds ratio of 2.61 and 1.88 in prevalence and incidence studies respectively [36]. Another meta-analysis of prospective studies found that higher education had a dose-response relationship with the decreased risk of dementia with a reduction in risk by 7% for per year increase in education level [35]. More importantly, several possible mechanisms underlying the protection against dementia associated with a higher education level have been proposed [33, 37, 38]. First, the “*reserve capacity*” hypothesis may explain the cognitive preservation among those with higher education [37]. Early education may have a direct impact on brain structure by boosting synapse quantity or vascularization, as well as establishing cognitive reserve [39]. Thus, early childhood education can slow the pace of cognitive decline in later life. Another explanation is the “*use it or lose it*” theory [38]. The population with higher education is more likely to continue searching for mental stimulation, resulting in postponing age-related cognitive decline [40]. Third, education in early life may affect late-life cognitive outcomes by changing a person’s SBDH [33]. For example, education affects a person’s occupation and health behaviors [33]. Interestingly, our findings revealed a complex interplay between education attainment, hypertension, *APOE4* gene, and dementia risk. Specifically, a decreased risk of dementia associated with college education or above was more pronounced among participants without hypertension, regardless of the presence of *APOE4* gene. While the exact mechanisms remain unclear, the association between hypertension and an increased risk of dementia has been well documented [41, 42]. Our results indicated that higher education levels may influence the lifestyle, potentially reducing the risk of hypertension and, consequently, dementia risk. Moreover, *APOE4* allele is a well-established genetic risk factor for Alzheimer’s disease, the most common form of demenia [43]. Notably, our study found that higher education levels may mitigate attenuate the *APOE4* gene-related dementia risk, indicating that education may serve as a modifiable factor capable of offsetting genetic predisposition. The observed protective effect of higher education against dementia, even in the presence of genetic risk factors, underscores the broader societal implications of our findings. It suggests that promoting education could have far-reaching effects on public health and cognitive aging. While these results are promising, future studies should focus on elucidating the mechanisms by which educational attainment reduces dementia risk and explore the complex interactions between education, hypertension, the *APOE4* gene, and risk of dementia.

Results from previous studies regarding the association between smoking and risk of dementia were mixed [15–17, 44]. Many studies found that smoking was significantly associated with an increased risk of dementia [16, 44–46], which is reinforced by the following plausible biological mechanisms: (1) smoking is a well-known risk factor for stroke [47], and thus may cause vascular dementia and AD [48]; and (2) smoking would adversely affect neurodegeneration through oxidative stress and inflammation [49] that were associated with increased production of amyloid-β and abnormal tau protein phosphorylation which are hypothesized to cause AD [50]. However, in our study, we found no significant association between current smoking and increased risk of all-cause dementia. Our results were consistent with one population-based longitudinal study which included 11,143 dementia-free participants aged 65 years and older [15]. The result showed that there was no significant association between smoking and the onset of all-cause dementia, AD, and vascular dementia during a mean of 3.8 years of follow-up [15]. We further explored the HTE of current smoking on dementia risk and identified key important covariates – men, history of cerebrovascular disease, and presence of neuropsychiatric disorders, that may modulate the association between smoking and dementia risk. Men typically have higher rates of cigarette smoking than women [51], which may partly explain the gender-specific effects observed in our study. Furthermore, the well-established link between smoking and increased risk of cerebrovascular disease [52], coupled with the known association between cerebrovascular disease and dementia [53], provides context for our HTE subgroup analysis findings. Specifically, we found that current smoking was significantly associated with an increased risk of dementia among men, with this effect being particularly pronounced in those with cerebrovascular disease. Neuropsychiatric disorders were associated with an increased risk of dementia, with differing impacts between men and women [54]. While further studies are warranted to fully understand the interaction between smoking, sex, cerebrovascular disease, neuropsychiatric disorders, and dementia, our research supports the potential benefits of smoking cessation programs in lowering dementia risk, even in older populations and especially among men with cerebrovascular conditions.

Prior research found detrimental effects of AUD on cognitive impairment and dementia [20, 55, 56]. One nationwide retrospective cohort study also found an increased risk of dementia associated with AUD among 19,769,440 adults (adjusted hazard ratio[aHR], 3.34 for women and 3.36 for men) [20]. Another cohort study involving 4,414 women veterans aged more than 55 years showed that AUD was significantly associated with an increased risk of dementia (adjusted HR, 3.12; 95%CI, 1.90–5.12) during a median follow-up of 4 years [55]. However, in our study, we found no association between AUD and all-cause dementia risk. Our single decision tree model identified neuropsychiatric disorders and men as the two key covariates that may affect the association between AUD and risk of all-cause dementia. These findings provide valuable insights into the complex interplay between AUD, neuropsychiatric disorders, and dementia risk. Neuropsychiatric disorders have been emerged as critical factors for development of all-cause dementia [57]. Simultaneously, there is a known association between AUD and risk of neuropsychiatric disorders, including depression, anxiety, and other psychiatric conditions [58]. The co-occurrence of AUD and neuropsychiatric disorders may create a synergistic effect, potentially exacerbating the risk of dementia. Our HTE subgroup analysis provided further nuance to these relationships. Specifically, we found that AUD was significantly associated with an increased risk of dementia in men who also had neuropsychiatric disorders. This finding suggests a potentially complex interaction between alcohol use, gender, mental health, and cognitive outcomes. Previous research has shown that neuropsychiatric disorders are associated with an increased risk of dementia, with differing impacts between men and women [54]. Additionally, men are more likely to smoke and have AUD, which may partly explain our study findings. Our findings suggest that individuals, particularly men, with both AUD and neuropsychiatric disorders may be at especially high risk for developing dementia. This underscores the need for integrated care approaches that address both alcohol use and mental health concerns as part of a comprehensive strategy to prevent cognitive decline. However, the HTE observed in our study also highlights the need for continued research to confirm and fully elucidate these relationships. Further investigation is warranted to better understand the complex interactions between AUD, neuropsychiatric disorders, gender, and dementia risk, and to develop more targeted prevention and intervention strategies.

This study has several advantages. First, we employed an assumption-free approach to uncover the potential HTEs of exposure of interest on the risk of all-cause dementia. This method overcomes the limitations of conventional ‘one-variable-at-a-time’ analysis, such as spurious findings and multiple hypotheses testing [59]. Second, the inclusion of detailed data on each exposure and precise dementia diagnosis enhances the overall quality of this study. However, we also acknowledge several limitations. First, as with any observational study, we cannot rule out residual confounding despite implementing advanced statistical techniques and adjusting for a list of covariates. Second, the NACC dataset is a clinic-based sample that is subjected to selection bias. The results were derived from the subjects referred by clinicians, patients, or family members, active recruitment, or volunteering, limiting the generalizability of our findings to the general population. Additionally, our sample was restricted to older adults, limiting generalizability to younger age groups. Third, smoking status, AUD, and other variables were derived from self-reported medical history, which may introduce uncertainty about the actual status and disease and potential recall bias or misclassification. It should be noted that our measure of AUD was based on a single item from the NACC questionnaire, which may not fully capture all aspects of AUD as defined by clinical diagnostic criteria such as those in the DSM-5 [60]. This limitation may have led to an underestimation of the participants with AUD in our sample and could have affected our findings regarding the association between AUD and dementia risk. Fourth, our study did not account for smoking severity and duration, which may contribute differentially to dementia risk [61]. In this study, smoking was classified into two categories (current smokers vs. all others), whereas future studies should consider more nuanced classifications. More nuanced classifications of smoking status should be considered in future studies. Similarly, educational level as a continuous variable rather than a categorical variable in future studies could offer additional insights into their association with dementia risk. Fifth, this study was limited by the small number of participants with AUD (37 out of 10,062) and current smoking (341 out of 10,062). These small sample sizes resulted in wider CIs, potentially accounting for the lack of significant association between these factors and risk of dementia in the overall cohort. Additionally, these limited sample sizes may lead to overfitting of our models for both AUD and current smoking. The findings for AUD and current smoking should be interpreted cautiously and require validation through further studies with larger sample sizes. Sixth, we included participants with normal cognition at baseline. This approach helps ensure that all participants started with relatively similar cognitive functions, reducing the likelihood that cognitive decline was influencing smoking or AUD at the study’s outset. However, subtle cognitive changes could still precede diagnosis by many years and potentially impact these behaviors. Finally, lifestyle factors, such as physical activity and diet, play a significant role in the development of dementia [62–64]. However, such factors are unavailable in NACC dataset, precluding a comprehensive analysis of the association between these lifestyle factors and risk of dementia in this study.

In conclusion, our study shows that participants with college education or above had a lower risk of all-cause dementia, especially among those without hypertension. While our analysis of the full cohort did not find a significant association between either current smoking or AUD and risk of all-cause dementia, we identified key variables that would contribute to their HTEs on dementia risk. These findings underscore the importance of adopting a personalized approach to addressing disparities in dementia care and prevention. Future research is warranted to confirm our findings and investigate the underlying mechanisms that drive the observed HTEs.

## Electronic supplementary material

Below is the link to the electronic supplementary material.

Supplementary Material 1

## Data Availability

The deidentified participant data could be requested through the National Alzheimer’s Coordinating Center (https://naccdata.org/requesting-data/data-request-process).
