# Supplementary material for: Association of education attainment, smoking status, and alcohol use disorder with dementia risk in older adults: a longitudinal observational study
Source: Alzheimers Res Ther. 2024 Sep 18;16:206. doi: 10.1186/s13195-024-01569-7 (PMC11412035; doi:10.1186/s13195-024-01569-7)
Supplement: Supplementary file 1 — Supplementary Material 1 [file 13195_2024_1569_MOESM1_ESM.docx]

**Supplementary materials**

**Table S1.** Baseline characteristics of participants stratified by college education or above

|  | **All, n (%)** | **College education or above, n (%)** | | **Standardized mean difference*** | |
| --- | --- | --- | --- | --- | --- |
|  |  | **Yes** | **No** | **Unweighted** | **Weighted** |
| No. of participants | 10,062 | 6390 | 3672 |  |  |
| **Sociodemographic** | | | | | |
| Age, >=80 yrs | 2619(26.0) | 1551(24.3) | 1068 (29.1) | -0.109 | 0.004 |
| Sex, men % | 3570 (35.5) | 2633(41.2) | 937(25.5) | 0.337 | 0.000 |
| Hispanic/Latino ethnicity | 559 (5.6) | 176(2.8) | 383(10.4) | -0.313 | 0.004 |
| Race, Black | 1429 (14.2) | 627(9.8) | 802(21.8) | -0.334 | 0.003 |
| Family history of dementia | 5165 (51.3) | 3383(52.3) | 1782(48.5) | 0.088 | 0.005 |
| *APOE4* carrier | 2546 (25.3) | 1637(25.6) | 909(24.8) | 0.020 | -0.008 |
| **Health Behaviors** | | | | | |
| Current smoking | 341 (3.4) | 161(2.5) | 180(4.9) | -0.126 | 0.001 |
| Alcohol use disorder | 37 (0.4) | 23(0.4) | 14(0.4) | -0.004 | -0.007 |
| **Comorbidities** | | | | | |
| Cardiovascular disease | 2537 (25.2) | 1591(24.9) | 946(25.8) | -0.020 | -0.002 |
| Cerebrovascular disease | 661 (6.6) | 377(5.9) | 284(7.7) | -0.073 | 0.005 |
| Neurological diseases | 777 (7.7) | 533(8.3) | 244(6.6) | 0.065 | -0.004 |
| Neuropsychiatric disorders | 2555 (25.4) | 1653(25.9) | 902(24.6) | 0.030 | 0.002 |
| Diabetes | 1212 (12.1) | 623(9.8) | 589(16.0) | -0.189 | 0.001 |
| Hypercholesterolemia | 5156 (51.2) | 3208(50.2) | 1948(53.1) | -0.057 | -0.004 |
| Hypertension | 5271 (52.4) | 3055(47.8) | 2216(60.4) | -0.254 | 0.010 |
| Obesity | 781 (23.3) | 1239(19.4) | 1105(30.1) | -0.250 | 0.001 |

* After inverse probability of treatment weighting (IPTW) with a standardized mean difference (SMD) ≤ 0.1 indicating a balance between the 2 groups.

Cardiovascular disease consists of heart attack/cardiac arrest, angioplasty/endarterectomy/stent, cardiac bypass procedure, pacemaker and/or defibrillator, congestive heart failure, atrial fibrillation, angina, heart valve replacement or repair, and other cardiovascular diseases;

Cerebrovascular disease included stroke and transient ischemic attack;

Neurological disease involves Parkinson’s disease (PD), other PD disorders, traumatic brain injury, seizures, and other neurological conditions;

Neuropsychiatric disorders include post-traumatic stress disorder, bipolar disorder, schizophrenia, depression, anxiety, obsessive-compulsive disorder, developmental neuropsychiatric disorders, and other psychiatric disorders.

**Table S2.** The final parameter set of propensity score model and outcome regression model.

| **Exposure of interest** | **Propensity score model** | **Outcome regression model** |
| --- | --- | --- |
| **College education or above** | LogisticRegression(C=0.1, random_state=13)” | LASSO (alpha=0.001, max_iter=500, normalize=False, random_state=13) |
| **Current smoking** | [LogisticRegression(C=5.0, random_state=13) | LASSO(alpha=0.0001, max_iter=500, normalize=False, random_state=13, selection='random') |
| **Alcohol use disorder** | LogisticRegression(C=0.01, random_state=13) | LASSO(alpha=1e-05, max_iter=500, normalize=True, random_state=13) |

**Table S3.** Performance metrics of the final model.

| **Exposure of interest** | **Final model^1^** | |
| --- | --- | --- |
|  | **training set** | **test set** |
| **College education or above** | 0.4 | 0.4 |
| **Current smoking** | 5.2 | 8.8 |
| **Alcohol use disorder** | 16.7 | 22.1 |

^1^ score based on the final stage loss (a lower score is better)

**Table S4.** Baseline characteristics of the participants stratified by current smoking

|  | **All, n (%)** | **Current smoking, n (%)** | | **SMD*** | | |
| --- | --- | --- | --- | --- | --- | --- |
|  |  | **Yes** | **No** | **Unweighted** | **Weighted** | |
| No. of participants | 10,062 | 341 | 9721 |  | |  |
| **Sociodemographic** | | | | | | |
| Age, ≥80 yrs | 2619(26.0) | 53(15.5) | 2566(26.4) | -0.269 | | 0.009 |
| Sex, men % | 3570 (35.5) | 122(35.8) | 3448(35.5) | 0.006 | | -0.009 |
| Educational level, ≥ college education | 6390 (63.5) | 161(47.2) | 6229(64.1) | -0.344 | | 0.031 |
| Hispanic/Latino ethnicity | 559 (5.6) | 20(5.9) | 539(5.5) | 0.014 | | 0.017 |
| Race, Black | 1429 (14.2) | 92(27.0) | 1337(13.8) | 0.333 | | -0.010 |
| Family history of dementia | 5165 (51.3) | 168(49.3) | 4997(51.4) | -0.043 | | -0.052 |
| *APOE4* carrier | 2546 (25.3) | 88(25.8) | 2458(25.3) | 0.012 | | 0.054 |
| **Health Behavior** | | | | | | |
| Alcohol use disorder | 37 (0.4) | 6(1.8) | 31(0.3) | 0.142 | | 0.000 |
| **Comorbidities** | | | | | | |
| Cardiovascular disease | 2537 (25.2) | 87(25.5) | 2450(25.2) | 0.007 | | -0.021 |
| Cerebrovascular disease | 661 (6.6) | 27(7.9) | 634(6.5) | 0.054 | | 0.013 |
| Neurological diseases | 777 (7.7) | 33(9.7) | 744(7.7) | 0.072 | | -0.002 |
| Neuropsychiatric disorders | 2555 (25.4) | 113(33.1) | 2442(25.1) | 0.177 | | -0.055 |
| Diabetes | 1212 (12.1) | 50(14.7) | 1162(12.0) | 0.080 | | 0.003 |
| Hypercholesterolemia | 5156 (51.2) | 180(52.8) | 4976(51.2) | 0.032 | | 0.007 |
| Hypertension | 5271 (52.4) | 199(58.4) | 5072(52.2) | 0.125 | | 0.029 |
| Obesity | 781 (23.3) | 78(22.9) | 2266(23.3) | -0.010 | | 0.035 |

* After inverse probability of treatment weighting (IPTW) with a standardized mean difference (SMD) ≤ 0.1 indicating a balance between the 2 groups.

Cardiovascular disease consists of heart attack/cardiac arrest, angioplasty/endarterectomy/stent, cardiac bypass procedure, pacemaker and/or defibrillator, congestive heart failure, atrial fibrillation, angina, heart valve replacement or repair, and other cardiovascular diseases;

Cerebrovascular disease included stroke and transient ischemic attack;

Neurological disease involves Parkinson's disease (PD), other PD disorders, traumatic brain injury, seizures, and other neurological conditions;

Neuropsychiatric disorders include post-traumatic stress disorder, bipolar disorder, schizophrenia, depression, anxiety, obsessive-compulsive disorder, developmental neuropsychiatric disorders, and other psychiatric disorders.

**Table S5.** Baseline characteristics of the participants stratified by Alcohol use disorder

|  | **All, n (%)** | **Alcohol use disorder, n (%)** | | **SMD*** | |
| --- | --- | --- | --- | --- | --- |
|  |  | **Yes** | **No** | **Unweighted** | **Weighted** |
| No. of participants | 10,062 | 37 | 10025 |  |  |
| **Sociodemographic** | | | | | |
| Age, ≥80 yrs | 2619(26.0) | 7(19.0) | 2612(26.1) | -0.171 | 0.099 |
| Sex, men % | 3570 (35.5) | 21(56.8) | 3549(35.4) | 0.436 | -0.017 |
| Educational level, ≥ college education | 6390 (63.5) | 23(62.2) | 6367(63.5) | -0.028 | 0.009 |
| Hispanic/Latino ethnicity | 559 (5.6) | 1(2.7) | 558(5.6) | -0.144 | -0.154 |
| Race, Black | 1429 (14.2) | 3(8.1) | 1426(14.2) | -0.194 | -0.026 |
| Family history of dementia | 5165 (51.3) | 25(67.6) | 5140(51.3) | 0.334 | 0.084 |
| *APOE4* carrier | 2546 (25.3) | 10(27.0) | 2536(25.3) | 0.039 | 0.077 |
| **Health Behaviors** | | | | | |
| Current smoking | 341 (3.4) | 6(16.2) | 335(3.3) | 0.439 | 0.047 |
| **Comorbidities** | | | | | |
| Cardiovascular disease | 2537 (25.2) | 9(24.3) | 2528(25.2) | -0.021 | **0.281** |
| Cerebrovascular disease | 661 (6.6) | 3(8.1) | 658(6.6) | 0.059 | -0.019 |
| Neurological diseases | 777 (7.7) | 4(10.8) | 773(7.7) | 0.106 | **0.249** |
| Neuropsychiatric disorders | 2555 (25.4) | 17(50.0) | 2538(25.3) | 0.438 | -0.020 |
| Diabetes | 1212 (12.1) | 9(24.3) | 1203(12.0) | 0.321 | **0.197** |
| Hypercholesterolemia | 5156 (51.2) | 16(43.2) | 5140(51.3) | -0.160 | -0.043 |
| Hypertension | 5271 (52.4) | 22(59.5) | 4249(52.4) | 0.142 | -0.037 |
| Obesity | 781 (23.3) | 8(21.6) | 2336(23.3) | -0.040 | -0.095 |

* After inverse probability of treatment weighting (IPTW) with a standardized mean difference (SMD) ≤ 0.1 indicating a balance between the 2 groups.

Cardiovascular disease consists of heart attack/cardiac arrest, angioplasty/endarterectomy/stent, cardiac bypass procedure, pacemaker and/or defibrillator, congestive heart failure, atrial fibrillation, angina, heart valve replacement or repair, and other cardiovascular diseases;

Cerebrovascular disease included stroke and transient ischemic attack;

Neurological disease involves Parkinson's disease (PD), other PD disorders, traumatic brain injury, seizures, and other neurological conditions;

Neuropsychiatric disorders include post-traumatic stress disorder, bipolar disorder, schizophrenia, depression, anxiety, obsessive-compulsive disorder, developmental neuropsychiatric disorders, and other psychiatric disorders.
